# Comparison of a Novel Ultra-Widefield Three-Color Scanning Laser Ophthalmoscope to Other Retinal Imaging Modalities in Chorioretinal Lesion Imaging

**DOI:** 10.1167/tvst.14.1.11

**Published:** 2025-01-13

**Authors:** Ines D. Nagel, Anna Heinke, Akshay P. Agnihotri, Shaden Yassin, Lingyun Cheng, Andrew S. Camp, Nathan L. Scott, Fritz Gerald P. Kalaw, Shyamanga Borooah, Dirk-Uwe G. Bartsch, Arthur J. Mueller, Nehal Mehta, William R. Freeman

**Affiliations:** 1 Jacobs Retina Center, Shiley Eye Institute, University of California San Diego, La Jolla, CA, USA; 2 Viterbi Family Department of Ophthalmology and Shiley Eye Institute, University of California San Diego, La Jolla, CA, USA; 3 Department of Ophthalmology, University Hospital Augsburg, Augsburg, Germany; 4 Division of Ophthalmology Informatics and Data Science, Viterbi Family Department of Ophthalmology and Shiley Eye Institute, University of California San Diego, La Jolla, CA, USA

**Keywords:** multicolor imaging, fundus photography, choroidal pigmented lesion, pseudocolor

## Abstract

**Purpose:**

To compare the assessment of clinically relevant retinal and choroidal lesions as well as optic nerve pathologies using a novel three-wavelength ultra-widefield (UWF) scanning laser ophthalmoscope with established retinal imaging techniques for ophthalmoscopic imaging.

**Methods:**

Eighty eyes with a variety of retinal and choroidal lesions were assessed on the same time point using Topcon color fundus photography (CFP) montage, Optos red/green (RG), Heidelberg SPECTRALIS MultiColor 55-color montage (MCI), and novel Optos red/green/blue (RGB). Paired images of the optic nerve, retinal, or choroidal lesions were initially diagnosed based on CFP imaging. The accuracy of the imaging was then evaluated in comparison to CFP using a grading scale ranging from –1 (losing imaging information) to +1 (gaining imaging information).

**Results:**

Eighty eyes of 43 patients with 116 retinal or choroidal pathologies, as well as 59 eyes with optic nerve imaging using CFP, MCI, RG, and RGB, were included in this study. Across all subgroups, RGB provided significantly more accurate clinical imaging with CFP as ground truth and compared to other modalities. This was true comparing RGB to both RG (*P* = 0.0225) and MCI (*P* < 0.001) overall. Although RGB provided more accurate clinical information overall, it was inferior to RG for melanocytic choroidal lesions (*P* = 0.011).

**Conclusions:**

RGB can be considered as a useful tool to detect characteristics of central, midperipheral, and peripheral retinal lesions. Regarding melanocytic choroidal lesions, RGB was inferior to RG, and MCI was inferior to both RG and RGB modalities due to color changes.

**Translational Relevance:**

Traditional retinal ultra-widefield imaging uses two wavelengths. Here, we evaluated three wavelengths for ultra-widefield imaging. We examined new optics (basic science) effect on patient imaging (clinical care).

## Introduction

Standardized assessment of choroidal or retinal lesions is important for initial ophthalmologic consultation, as well as follow-up consultations. White-flash color fundus photography (CFP) has been the standard of care for fundus imaging and documenting retinal and choroidal lesions because the color rendition is similar to that seen by clinicians using ophthalmoscopy.^1^^–^^4^ However, CFP can be uncomfortable due to the bright flash and requires dilation for peripheral retinal view.^5^^–^^7^ More recently, ultra-widefield (UWF) pseudocolor imaging has become commonly used and has begun to replace CFP in daily practice.

UWF allows rapid, undilated 200° UWF imaging.^8^ The Optos California Ultra-Widefield Retinal Imaging System (red/green [RG]; Optos, Dunfermline, UK) uses red (633-nm) and green (532-nm) laser wavelengths, which can lead to red-tinged imaging.^9^^–^^11^ Other devices from different manufacturers offer the ability to perform tracked autofluoresence, spectral-domain optical coherence tomography, and fundus imaging using scanning laser ophthalmoscopes that include three different wavelengths (blue, 488 nm; green, 514 nm; and infrared, 815 nm) as part of multicolor imaging (MCI).^12^^,^^13^ However, this system is limited because it does not provide an UWF image and the pseudocolor image is also noted to be different from that of white-light fundus photography.^12^^,^^14^ Recently, an updated UWF imaging device was launched that uses red (633-nm), green (532-nm), and blue (488-nm) (RGB) wavelengths to leverage the advantages of undilated UWF imaging in an attempt to render an image closer to CFP imaging. After acquiring the UWF image, a postprocessing image rendering takes place. The exact rendering algorithm is proprietary.

This study examined whether RGB is superior to RG or MCI using CFP as a reference to diagnose retinal and choroidal lesions. Additionally, the research sought to establish if particular imaging techniques outperform others in picturing specific chorioretinal and optic nerve diseases.

## Methods

This study was performed at the Department of Ophthalmology, Shiley Eye Institute, University of California San Diego (UCSD), between June 2023 and November 2023. Patients were included if they had been diagnosed with any tumor-like melanocytic choroidal lesion, drusen, choroidal neovascularization (CNV), retinal tears, bone spicule pigmentation, atrophic lesions, or hemorrhages. Institutional Review Board approval from UCSD was obtained for the retrospective and prospective review of patients’ charts and images (approval no. 120516). Patients were excluded if retinal pathology could not be clearly seen in any imaging. The study adhered to the tenets of the Declaration of Helsinki for research involving human subjects and complied with Health Insurance Portability and Accountability Act regulations.

As our clinical standard, all patients underwent multimodal imaging on the same day after pupil dilation. Color fundus photography was taken with the Topcon TRC50X digital fundus camera (Topcon, Tokyo, Japan), and multicolor images were taken using SPECTRALIS HRA-OCT (Heidelberg Engineering, Heidelberg, Germany). For the devices, a 55° lens and a 50° lens were used. Steered images were used to visualize peripheral pathologies on each imaging device. Additional fundus photography was performed with the Optos California RG and California RGB. Pupil dilation was performed with phenylephrine 2.5% and tropicamide 1% and a wait of at least 20 minutes. Patients with missing imaging of one or more devices were excluded.

All fundus images were deidentified before the analysis. The images were saved as maximum quality portable network graphic (PNG) image files with no modifications. In each imaging, an area of pathology was identified. Each pathology per image set was selected, and lesion borders were adjusted to show the same borders and angles of pathology in each imaging modality. Each pathology was cropped to a circle using Photoshop (Adobe, San Jose, CA).

**Figure 1.**
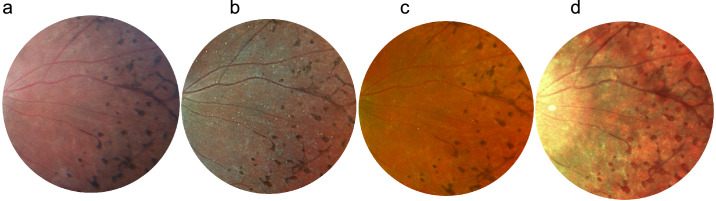
Multicolor images and CFP of bone spicules. Bone spicules are visible in all modalities: CFP (**a**), RGB (**b**), RG (**c**), MCI (**d**).

**Figure 2.**
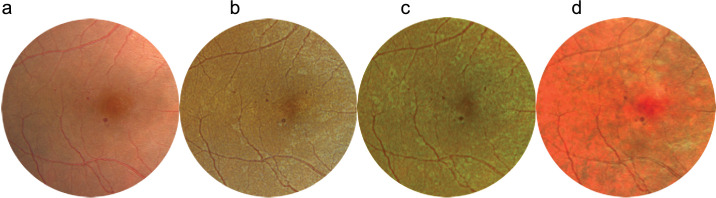
Multicolor images and CFP of intraretinal hemorrhages. The hemorrhages are well differentiated in CFP (**a**) and RGB (**b**) compared to RG (**c**) and MCI (**d**).

**Figure 3.**
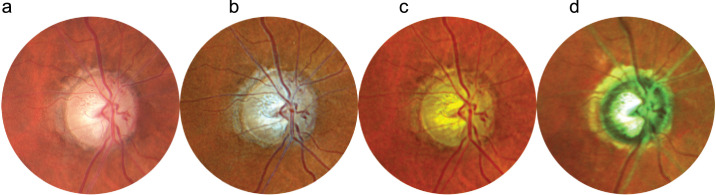
Multicolor images and CFP of a pale and cupped optic disc. (**a**) CFP offers true color. (**b**) RGB gives the impression of a pale optic nerve. (**c**) RG offers red-tinged imaging. (**d**) MCI offers false color with emphasis of the cup.

**Figure 4.**
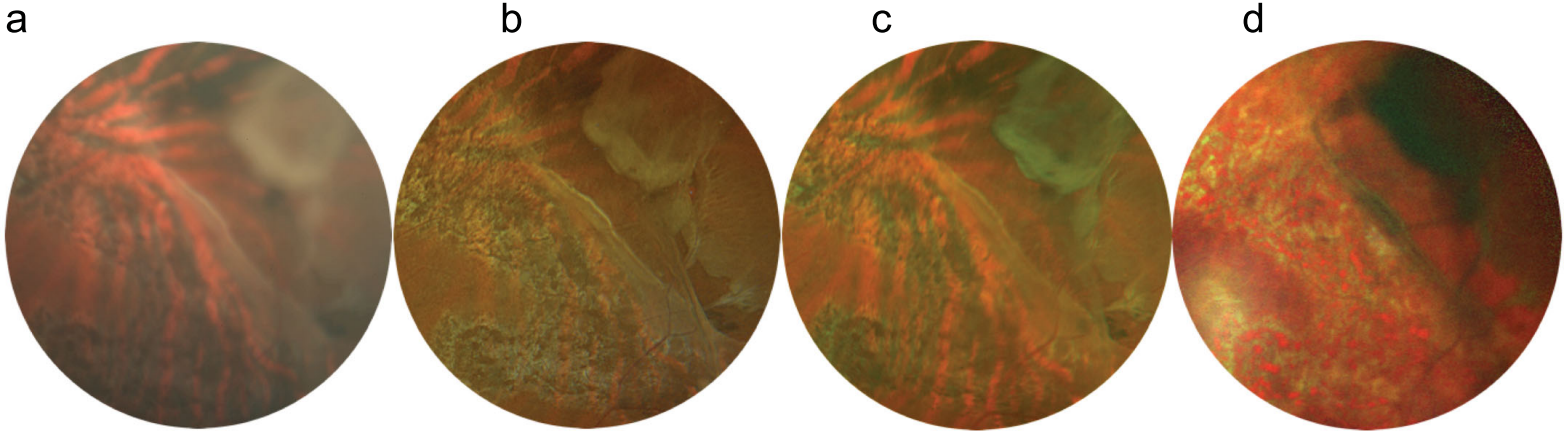
Peripheral retinal tear. (**a**–**c**) CFP (a) shows peripheral lesions less detailed as RGB (b) or RG (c). (**d**) MCI offers a different appearance due to color rendition.

**Figure 5.**
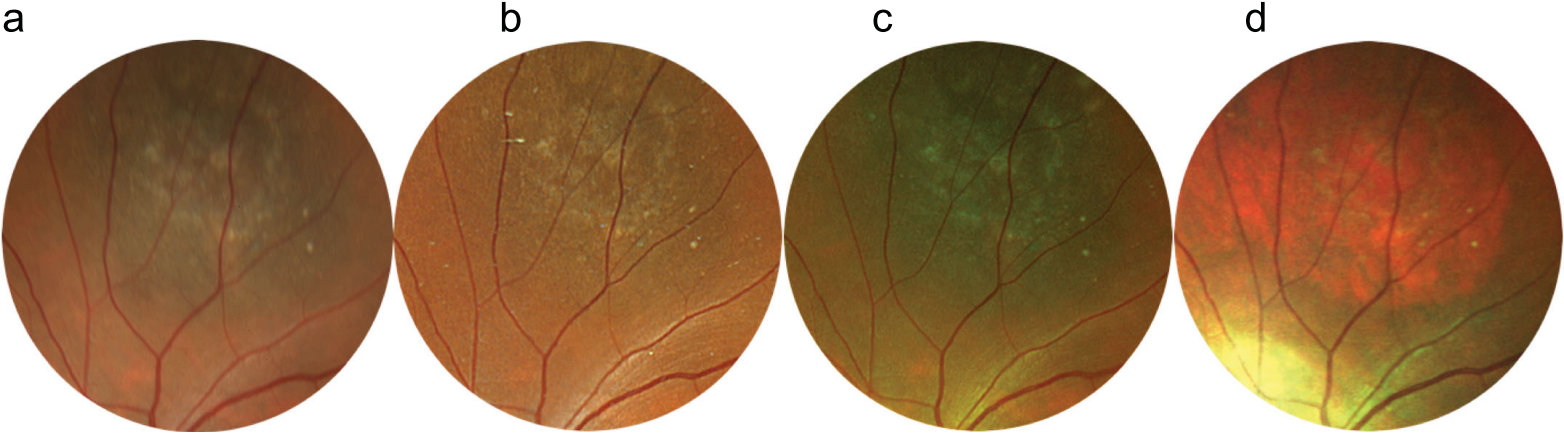
Multicolor images and CFP of a choroidal nevus. (**a**–**c**) The choroidal pigmentation is visible in CFP (a) and RG (c). The lesion is presented with unusual color rendition in RGB (b). (**d**) In MCI, the lesion appears red, and borders can be seen.

Pathologies were divided into five groups:

1. General midperipheral retinal and retinal pigment epithelium (RPE) pathologies (intraretinal hemorrhages, microaneurysms, retinal ischemia, crossing signs, myelinated nerve fibers, bone spicules) (Fig. 1)
2. Macular pathologies (drusen, CNV, macular hole, epiretinal membrane, geographic atrophy) (Fig. 2)
3. Optic disc pathologies (glaucoma, glaucoma suspect, waxy pallor due to retinal degeneration) (Fig. 3)
4. Focal peripheral lesions (retinal holes, peripheral degeneration) (Fig. 4)
5. Melanocytic choroidal lesions (nevus, choroidal melanoma) (Fig. 5)

**Figure 6.**
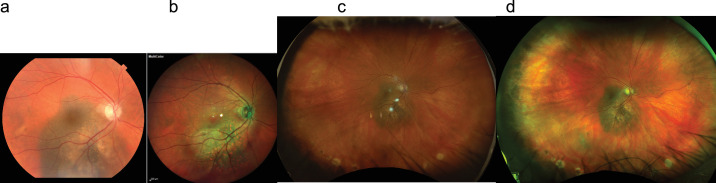
Multicolor images and CFP of a choroidal nevus before cropping. The choroidal pigmentation is visible in CFP (**a**) and RG (**d**). The lesion appears more pale in RGB (**c**). In MCI (**b**), the lesion appears red, and borders cannot be distinguished clearly.

An example of an unprocessed image set is shown in ^1^,^2^,^3^,^4^,^5^Figure 6.

We designed a classification framework for each group based on established protocols determining diabetic retinopathy and retinal multimodal imaging.^8^^,^^12^^,^^15^^–^^21^ Ten reference images were provided to train graders before grading. These images were excluded from the study set.

Two experienced retina specialists (APA, AH) graded each retina image at different time points in the same room under optimal room conditions. Identical computers and monitors were used, as well as user settings to avoid monitor-induced differences (Cinema HD, 100% brightness; Apple, Cupertino, CA). The optic disc pathologies were rated by a glaucoma specialist (ASC) with the above-mentioned classification. The graders were given the CFP image initially along with the clinical diagnosis based on ophthalmoscopy. Subsequently, the other imaging modalities were presented. The graders were then asked to grade the images with the mentioned groups based on the CFP image and the clinical diagnosis. The graders were blinded when grading the images from the RGB and RG machines.

### Grading

For each image set, examiners were asked to rate each image modality using a grading scale between –1 and +1 compared to the imaging that was first presented, which was the CFP:

• Grade –1—The lesion appeared different in the imaging modality compared to CFP imaging and the same diagnosis could not be made.
• Grade –0.5—The same diagnosis could be made in the imaging modality compared to CFP imaging with some loss of information.
• Grade 0—The same diagnosis could be made in the imaging modality compared to CFP imaging without any loss or gain of information.
• Grade +0.5—The same diagnosis could be made in the imaging modality compared to CFP imaging with a slight gain of information (e.g., improved clinical impression compared to CFP).
• Grade +1—The same diagnosis could be made with an obvious gain of information in the imaging modality compared to CFP imaging.^4^

A grade was confirmed after the retinal experts agreed. In case of disagreement, a meeting with a third retina specialist (IDN) was held to arrive at a consensus grading.

### Statistical Analysis

Image quality of a specific lesion of a patient was graded as five ordinal scores using CFP as a reference. Each lesion (patient) had three images in each three image modalities. To compare image modalities (RGB, RG, or MCI) for better visibility of the lesion, a repeated ordinary regression was performed using scores as the response and image modality and lesion type as independent variables. In this multivariate regression analysis, age and gender as important biological parameters were adjusted. For optic nerve imaging, image modality of RGB was compared with RG for superiority of the images using paired *t*-tests. The analysis was performed using SAS 9.4 (SAS Institute, Cary, NC).

## Results

**Figure 7.**
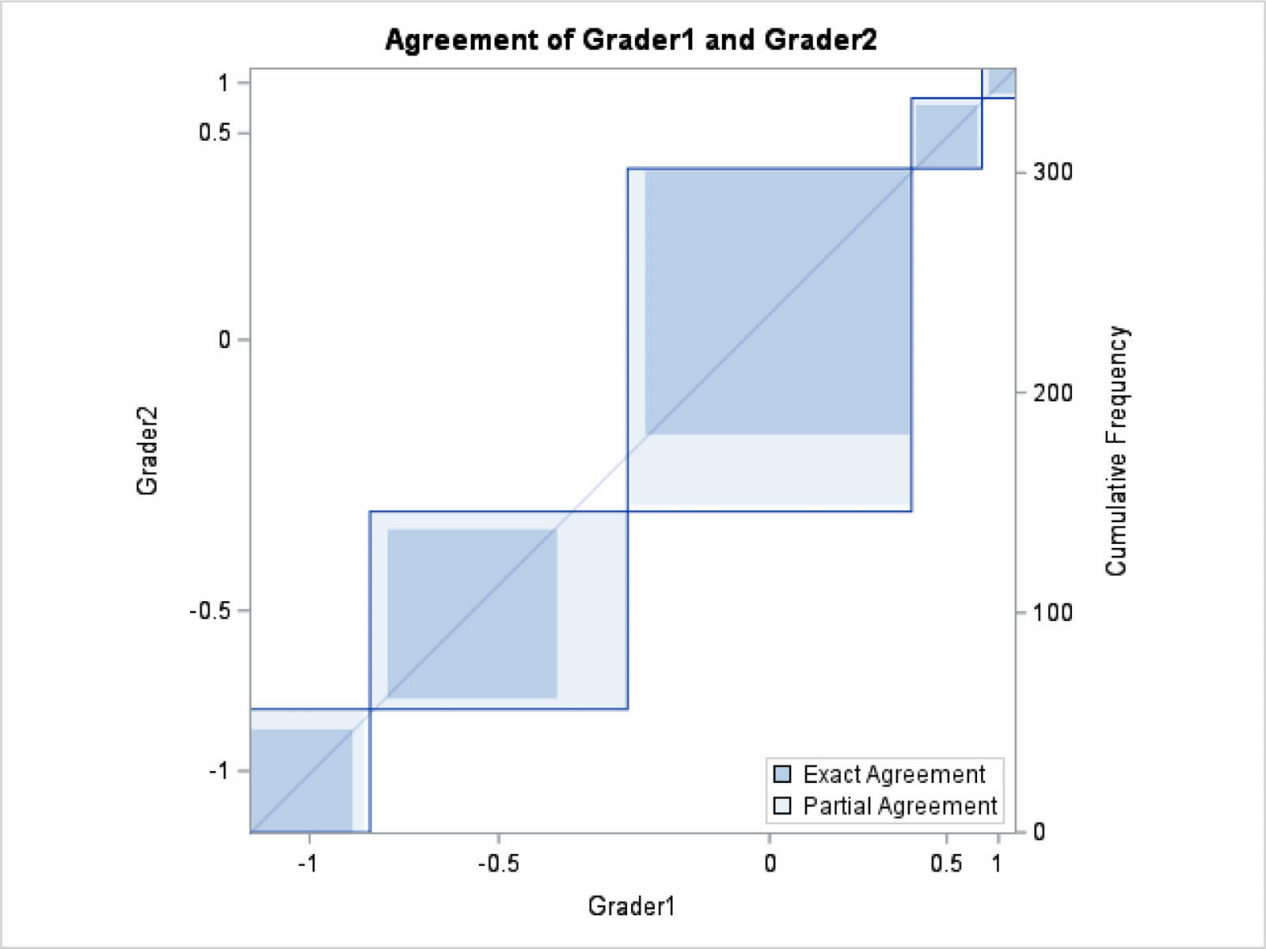
Agreement between grader 1 and grader 2. CFP, Topcon CFP; RGB, Optos UWF RGB wavelengths; RG, Optos UWF RG wavelengths; MCI, Heidelberg SPECTRALIS multicolor imaging.

Sixty-six patients were initially identified, 23 of whom had insufficient image quality and hence were excluded. A total of 80 eyes from 43 patients were included in the study; 116 retinal and choroidal imaging sets with pathology were identified in the study, and 59 sets of optic nerves were included. The baseline characteristics of the patients are shown in Table 1, the distribution of pathologies is shown in Table 2, and the grading scores are shown in Table 3 for RGB, RG, and MCI. The grading system had a high reliability with an intergrader weighted kappa of 0.82 (95% confidence interval, 0.77–0.86; test of weighted kappa = 0; *P* < 0.0001). The agreement of graders is shown in Figure 7.

**Table 1.** Baseline Characteristics

Characteristic	Value
Age, (y) mean ± SD (range)	59.0 ± 18.94 (18–97)
Gender (female/male), *n*	19/24
Pathologies, *n*	
Group 1 (midperipheral retinal/RPE changes)	26
Group 2 (macular pathologies)	48
Group 3 (optic nerve)	59
Group 4 (focal peripheral lesions)	17
Group 5 (pigmented melanocytic lesions)	25

**Table 2.** Distribution of Pathologies

Pathology	*n*
Age-related macular degeneration (drusen, geographic atrophy, CNV, subretinal hemorrhage)	25
Epiretinal membrane	8
Inherited macular dystrophy	6
Cotton wool spots	3
Microaneurysms, neovascularization elsewhere, intraretinal hemorrhage	12
Pigment mottling, congenital hypertrophy of the RPE	11
Retinal tear	8
Bone spicules	7
Myelinated fibers	2
Pigmented choroidal lesion	25
Peripheral chorioretinal scar (laser, surgery, degeneration)	9

**Table 3.** Grading RGB, RG, and MCI Compared to CFP

	Grade				
	−1	−0.5	0	+0.5	+1
Group 1					
RGB (midperiphery)	0%	4%	73%	19%	4%
RG (midperiphery)	0%	23%	69%	8%	0%
MCI (midperiphery)	8%	62%	30%	0%	0%
Group 2					
RGB (macula)	2%	17%	62%	9%	9%
RG (macula)	4%	17%	62%	11%	2%
MCI (macula)	40%	28%	26%	2%	0%
Group 3					
RGB (optic disc)	10%	27%	63%	0%	0%
RG (optic disc)	22%	51%	27%	0%	0%
MCI (optic disc)	63%	32%	5%	0%	0%
Group 4					
RGB (periphery)	6%	6%	29%	29%	29%
RG (periphery)	0%	18%	35%	29%	18%
MCI (periphery)	24%	65%	6%	6%	0%
Group 5					
RGB (melanocytic)	16%	40%	44%	25%	0%
RG (melanocytic)	0%	36%	60%	4%	0%
MCI (melanocytic)	92%	4%	4%	0%	0%

RGB was found to provide greater clinical image information using CFP as reference in 23% of eyes in Group 1 (6/26), in 18% of eyes in Group 2 (8/47), and in 59% of eyes in Group 4 (10/17). It provided comparable or no additional information in Groups 3 and 5. RGB provided lesser information compared to CFP in 0% of eyes in Group 1 (0/26), 2% in Group 2 (1/47), 10% in Group 3 (6/59), 6% in Group 4 (1/17), and 16% in Group 5 (4/25) (Table 2). Within Group 3, RGB showed a false pallor of the optic nerve.

RG was found to provide more image information using CFP as reference in 8% within Group 1 (2/26), in 13% within Group 2 (6/47) and in 59% within Group 4 (10/17). It gave equal or no additional information in Groups 3 and 5. RG gave less information compared to CFP for 0% in Group 1 (0/26), 4% in Group 2 (2/47), and 22% in Group 3 (13/59). No information was lost in Groups 4 and 5 (Table 2).

MCI provided the same quality image information using CFP as reference in 30% within Group 1 (8/26), in 26% within Group 2 (12/47), in 5% within Group 3 (3/59), in 6% within Group 4 (1/17), and 4% in Group 5 (1/25). The least information loss with CFP as ground truth was noted in midperipheral lesions (8%, 2/26). The greatest information loss was noted in melanocytic choroidal lesions (92%, 23/25) (Table 2). Confounding factors (age and gender) did not show a significant influence on the results (*P* = 0.6087 and *P* = 0.8136, respectively).

The following observations emerged from the summary of all groups (Groups 1–5): In pairwise analyses of scoring, using the different modalities, RGB provided the most similar images of pathology, when compared to CFP gold standard, against MCI (*P* < 0.001) and RG (*P* = 0.0225). RG overall presented significantly more similar clinical imaging compared to MCI (*P* < 0.001).

The following information was recorded within the individual pathology groups for RGB and RG: In Group 1 (general RPE and retinal pathologies, *n* = 26), RGB provided significantly higher similarity in imaging compared to UWF RG (*P* = 0.0021) with CFP baseline. In Group 2 (macular pathologies, *n* = 47), RGB provided significantly higher similarity in imaging compared to RG (*P* = 0.0110) with CFP baseline. In Group 3 (optic nerve, *n* = 59), RGB provided significantly higher similarity in clinical imaging compared to RG (*P* < 0.0001) with CFP baseline. In Group 4 (focal peripheral lesions, *n* = 17), no significant differences were observed between RGB and RG (*P* = 0.2156). In Group 5 (melanocytic choroidal lesions, *n* = 25), RG provided significantly higher similarity in imaging compared to RGB (*P* = 0.011) with CFP baseline.

## Discussion

The most important finding is that RGB provides more similar clinical imaging information than RG and MCI when detecting macular lesions and general retinal pathologies. However, RGB provides less similarity to RG in imaging of melanocytic choroidal lesions due to unusual color alteration. MCI also showed a different lesion coloration of melanocytic lesions.

Standard color fundus white-flash photography remains the gold-standard imaging modality to document fundus findings in true color; however, it is a time-consuming imaging procedure that requires patients to be dilated and cooperative and is often uncomfortable due to multiple bright white flashes. Laser-based red and green UWF imaging can be used to capture a retinal view over 200° in pseudocolor. Laser-based red, green, and blue UWF imaging is a new retina imaging method that can be performed similarly to UWF red and green imaging in the same setting and with the same acquisition time.

This study was specifically designed to assess whether UWF Optos California RGB provides more similar clinical imaging compared to UWF Optos California RG or Heidelberg SPECTRALIS MCI with Topcon CFP baseline and whether it might be used as a standard method without information loss compared to UWF Optos RG. To the best of our knowledge, this study is the first to discuss the imaging differences of the Optos California UWF RGB compared to Optos California UWF RG, Heidelberg SPECTRALIS MCI, and Topcon CFP. In order to avoid location bias, the melanocytic lesions were studied in different locations of the retina. The melanocytic choroidal lesions presented in macula, midperiphery or the periphery, were all better seen on UWF RG compared to UWF RGB.

It is possible that the additional blue wavelength in UWF RGB alters the retinal documentation to the advantage of lesions such as drusen and to the disadvantage of melanocytic choroidal lesions, as well as the optic nerve imaging. This can be explained with the short blue wavelength being most accurate in imaging inner retinal structures such as the retinal nerve fiber layer, epiretinal membrane, and retinal folds.^22^ Despite the ability of the UWF RGB to provide the most similar images to color fundus photography, the tendency to brighten melanocytic choroidal lesions may not allow this imaging modality to be used as a first screening option of retinal pathologies or as a documentation tool of melanocytic choroidal lesions. It may be possible that there is an intensifying effect with melanocytic lesions in the UWF RG that is different in the UWF RGB. Nevertheless, in this study the UWF RGB showed images of retinal lesions that were most similar to CFP imaging.

When looking at macular and mid-peripheral lesions other than melanocytic choroidal lesions, the UWF RGB showed the most similar imaging to CFP. UWF RGB was not superior to UWF RG in peripheral retinal lesions such as retinal tears. It can be assumed that UWF RG already has accurately identified the lesions.

There have been limited reports on the utility of widefield retinal imaging with different modalities.^2^^,^^4^^,^^5^^,^^11^ To the best of our knowledge, this study is the first to discuss the optical findings of the Optos California UWF RGB compared to Optos California UWF RG, Heidelberg SPECTRALIS MCI, and Topcon CFP. A previous study reported that MCI underestimates the borders of pigmented choroidal lesions and shows a different lesion coloration.^12^ Hirano et al.^16^ were able to show that UWF RG is able to effectively document intraretinal pathologies. Different from these studies, we evaluated whether UWF RGB can be used to provide the same or more true-color-like information compared to UWF RG or MCI.

There are limitations to this study. Overall, images taken with CFP can look very different from UWF RGB, UWF RG, and MCI. Also, images taken with UWF RGB, UWF RG, and MCI can also look very different from one another.^12^ Even after layering and cropping into the same size and angle of view, it is not completely possible to mask the observers while grading. Also, the grader is likely to prefer the CFP because it presents the retinal findings closer to clinical indirect ophthalmoscopy. Nevertheless, by directly comparing the images, the graders were able to decide whether they could recognize additional information (e.g., clear borders of lesions, sharper peripheral lesions, more detailed information such as bridging vessel and orange pigment) in an image or whether they noticed a loss of information. Additionally, by viewing the images with a given diagnosis, there is a bias. However, this bias then exists for each subsequent image of the same pathology presented to the grader.

Because pigmentation of the choroidal lesions is important for diagnosis, it is unclear whether UWF RGB can be recommended as a sole retinal screening device or for follow-up documentation. Therefore, longitudinal studies with more patients are required to determine whether UWF RGB can be used either as a sole retinal screening tool or for follow-up documentation of melanocytic choroidal lesions. As a further examination, it would be advisable to carry out a comparison of both UWF modalities by general ophthalmologists to examine whether retinal pathologies are overlooked in different imaging modalities. It is yet to be determined how many retinal pathologies would be missed when looking at the full uncropped UWF images. In addition, it should be considered that the patient group is heterogeneous. Peripheral retinal lesions are more difficult to document with CFP, and image quality may be impacted.

## Conclusions

UWF RGB can provide more accurate clinical imaging information for specific pathologies. However, physicians should be aware of the altered color representation of melanocytic choroidal lesions when using the new UWF RGB.
